# Temperature effects on productivity, respiration, and dissolved organic matter degradation in subtropical Florida waters following hurricane-driven riverine runoff and brownification

**DOI:** 10.3389/fmicb.2026.1884823

**Published:** 2026-09-15

**Authors:** Bruna F. Sobrinho, Amanda Muni-Morgan, Cynthia A. Heil, Patricia M. Glibert

**Affiliations:** 1 Horn Point Laboratory, University of Maryland Center for Environmental Science, Cambridge, MD, United States; 2 College of Marine Science, University of South Florida, St. Petersburg, FL, United States; 3 Mote Marine Laboratory, Sarasota, FL, United States

**Keywords:** brownification, humic substances, hypoxia, primary production, respiration

## Abstract

Increasing ocean warming and storm-driven terrestrial inputs are reshaping subtropical coastal ecosystems. This study investigated how elevated temperature and humic acid enrichment, under both pre- and post-hurricane (Idalia, Debby, Helene and Milton) *in situ* and manipulated conditions, affect the metabolic rates of phytoplankton communities on the West Florida Shelf (WFS). We hypothesized that warming, combined with hurricane-driven humic inputs, would decrease primary production rates while enhancing respiration and dissolved organic matter (DOM) degradation, shifting system toward net heterotrophy. Using ^18^O techniques combined with oxygen concentration measurements, the differences between light-dependent respiration (hereafter Gross Respiration, GR, representing only that associated with algal respiration) and dark respiration (hereafter Community Respiration, CR, representing the sum of microbial respiration) were measured in addition to rates of Gross Primary Production (GPP), and Net Primary Production (NPP). Across stations and experiments, increasing temperature consistently reduced GPP and shifted NPP toward negative values due to enhanced GR. Rates of algal light-dependent GR compared favorably to total CR, in some cases exceeding CR. Changes in the Fluorescent Dissolved Organic Matter (FDOM) composition were also analyzed using spectrofluorometric methods. Increasing temperature was associated with enhanced biological production of labile, protein-like FDOM and concurrent shifts toward lower humification. Together, these changes increased oxygen demand while decreasing oxygen solubility, creating conditions favorable to hypoxia. These results show that warming and hurricane-driven brownification are associated with enhanced algal respiratory metabolism in addition to enhanced CR, highlighting a potential pathway through which climate change could intensify hypoxia in subtropical coastal systems.

## 1 Introduction

Ocean and coastal systems are increasingly being affected by climate change and the multiple environmental stressors related to it. Warming and nutrient pollution are among the most important drivers reshaping aquatic environments. Estuaries and coastal zones respond rapidly to these pressures, and by integrating terrestrial and marine influence act as sentinels of environmental changes (Cloern et al., 2016). At the same time, extreme weather events, including storms, heatwaves, and droughts, are also becoming more frequent and intense (Glibert and Li, 2024). Understanding the complexities of these overlapping interactions remain a major challenge (Bijma et al., 2013; Glibert et al., 2022; Eddy et al., 2025; Guild et al., 2025). Relationships between temperature increase, nutrient enrichment, hypoxia and harmful algal blooms (HABs) have received considerable attention (e.g., Anderson et al., 2008; Hallegraeff, 2010; Martínez et al., 2017; Gobler et al., 2017; Glibert, 2019; Griffith and Gobler, 2020; Glibert et al., 2020). However, less well understood are the effects of changing terrestrial dissolved organic matter (DOM) loads to coastal water due to changes in extreme weather events, riverine discharge, sea level rise and other factors, and how those loads are altering primary production and respiration. This is especially true in subtropical waters where organic loads and brownification have been poorly studied.

The increasing frequency and intensity of storms, hurricanes, and high riverine runoff associated with climate change are altering the delivery of nutrients and organic matter to coastal waters. During periods of intense runoff, allochthonous organic matter, primarily in the form of terrestrially derived, colored humic and fulvic acids, can dominate the DOM pool (Thurman, 1985; Moran and Hodson, 1994). Storm-driven turbulence further mobilizes sediments and detritus from nearshore vegetation, enhancing the export of organic matter and nutrients to coastal zones (e.g., Miles et al., 2013; Bianucci et al., 2018; Chen et al., 2019). In urbanized watersheds engineered drainage networks, increased impervious surface area, and wastewater effluent contribute additional loads of DOM including labile dissolved organic nitrogen (DON) that can stimulate HABs (Bronk et al., 2010; Kaushal and Belt, 2012). Although humic substances have traditionally been considered largely refractory (Opsahl and Benner, 1997; Benner, 2002; Coates et al., 2002), growing evidence shows that this pool, or a large fraction of this pool, can be biologically reactive, with implications for water quality, biogeochemical cycles, and the structure and functioning of planktonic communities (Stepanauskas et al., 2002; Wiegner et al., 2006; Boyer et al., 2006; See et al., 2006; Mendoza and Zika, 2014; Hounshell et al., 2022; Yin et al., 2025; Zhang et al., 2025).

A substantial fraction of DOM, up to 80% in freshwater, consists of dissolved humic substances, naturally colored, high-molecular-weight organic acids (Thurman, 1985). The enrichment of coastal waters with these materials, a process known as brownification, alters underwater light fields and can influence primary production and bloom dynamics (Urrutia-Cordero et al., 2017; Blanchet et al., 2022). In addition to their optical effects, humic substances can play an important role as metal chelators (e.g., Musani et al., 1980) and can also serve as a nutrient source; much of the nitrogen (N) they contain is in the form of loosely bound ammonium (NH_4_^+^) that can be released through photooxidation or ion-exchange processes as riverine waters mix with higher-salinity coastal waters (Bushaw et al., 1996; Bushaw-Newton and Moran, 1999; Cochlan and Bronk, 2001; See et al., 2006). Brownification can reduce light penetration while simultaneously enhancing heat absorption, creating warmer and darker conditions that may selectively favor taxa adapted to such environments (Williamson et al., 2015; Pilla et al., 2018). Several dinoflagellates, including *Prorocentrum minimum*, *Karenia brevis*, and *Pyrodinium bahamense*, have been shown to use humic-derived substrates (Granéli et al., 1999; Heil, 2005; See et al., 2006; Muni-Morgan et al., 2023; Zhang et al., 2025). However, the influence of humic enrichment combined with increasing temperature on phytoplankton metabolism, including both primary production and respiration, remains poorly understood especially in subtropical waters.

Warming and organic matter enrichment can enhance oxygen (O_2_) consumption in coastal waters (Breitburg et al., 2018). At the same time, O_2_ solubility decreases with increasing temperature and with the input of fresh waters (Schmidtko et al., 2017). Warmer conditions also promote higher water column stability and stratification, isolating water masses rich in organic matter and restricting vertical mixing (e.g., Lønborg et al., 2020; Goñi et al., 2021). Together, these processes stimulate microbial respiration processes, which rise with temperatures (Wikner et al., 2023), and contribute to the expansion and intensification of hypoxia regionally and globally (Weiss, 1970; Diaz and Rosenberg, 2008; Grégoire et al., 2021; Alam, 2023). Respiration has also been shown to increase with DOM loading (Pace et al., 2021). However, there are still many questions regarding how these rates of respiration contribute to hypoxia and how these rates may be influenced by different environmental factors. Importantly, with increased knowledge of the processes contributing to respiration, it is now clear that traditional methods of measuring respiration via changes in O_2_ (i.e., “light bottle-dark bottle” techniques, Gaarder and Gran, 1927) are insufficient to fully characterize those rates, as they only measure dark respiration. Phytoplankton also respire in the light, initiated through photosynthesis, via processes such as cyclic electron flow, the Mehler reaction, and photorespiration, which regulate cellular redox by dissipating excess energy, preventing photoinhibition caused by excess of light or downstream metabolic limitation (Kana, 1990; Lewitus and Kana, 1995; Kramer and Evans, 2011; Glibert et al., 2016 and references therein). However, understanding how rates of light-dependent respiration compare to microbial community respiration remains under quantified, especially under conditions of elevated DOM. Therefore, to fully constrain how DOM impacts all respiration processes, it is necessary to measure algal respiration rates, not just the rates of O_2_ consumption during microbial degradation.

The Gulf of Mexico (GoM; Gulf of America) provides an ideal setting to investigate the impacts of multiple environmental stressors related to climate change, including the increasing organic loads associated with hurricanes. The northern GoM is characterized by one of the largest regions of human-induced hypoxia in the world, and the largest along the U. S. coastline (Rabalais and Turner, 2019; Pontius and McIntosh, 2024). Surface water temperatures in the GoM have increased at nearly twice the rate observed in the global ocean surface (Wang et al., 2023).

Due to its location near the warm waters of the North Atlantic, including the Caribbean Sea, Florida experiences more hurricanes than any other U. S. state (Malmstadt et al., 2009, 2010; Gilliam, 2021). Notably, in 2023, Florida experienced Hurricane Idalia (August 30, 2023) and in 2024, Florida was impacted by three major hurricanes: Debby (August 3, 2024), Helene (September 26, 2024), and Milton (October 9, 2024). Hurricanes Idalia (Category 4) and Debby (Category 1) generated extreme rainfall across Florida’s west coast (National Weather Service, 2024), resulting in intense river flow and flooding, considerably enhancing freshwater discharge to coastal waters and the export of terrestrially derived sediments and organic matter to the coastal ocean. In contrast, Helene (Category 4), primarily a storm-surge–dominated event, produced less rainfall but generated record-high surge levels along Florida’s west coast due to strong onshore winds. This led to major coastal flooding (Florida State University Climate Center, 2024). Hurricane Milton (Category 5) combined extreme storm surge with heavy rainfall, resulting in a mixed forcing regime in which both surge-driven coastal erosion and rainfall-enhanced river discharge contributed to sediment resuspension and organic matter export to the coastal ocean (National Hurricane Center, 2024). Such extreme events can–and did–intensely alter the typical distribution of DOM on the West Florida Shelf (WFS) (e.g., Conmy et al., 2009; Mendoza and Zika, 2014).

A unique feature of the eastern GoM that can also contribute to local anoxia/hypoxia are the near-annual blooms of the toxic dinoflagellate *Karenia brevis*. These blooms can be sustained by hurricanes and tropical storms which deliver large pulses of nutrients and organic matter, sometimes comparable to the entire annual nutrient load to the region (Hu et al., 2006; Fritz, 2018; Chen and Li, 2025). Studies have reported the presence of hypoxia during intense *K. brevis* blooms (Smith, 1975; Heyl et al., 1978; Milbrandt et al., 2021; Turley et al., 2022). Recently it has been hypothesized that hypoxia occurring in parallel to a *K. brevis* red tide event is more likely to occur with warmer ocean temperatures and increased fluxes of organic nutrients, especially after hurricanes (Milbrandt et al., 2021; Figure 1).

**Figure 1:**
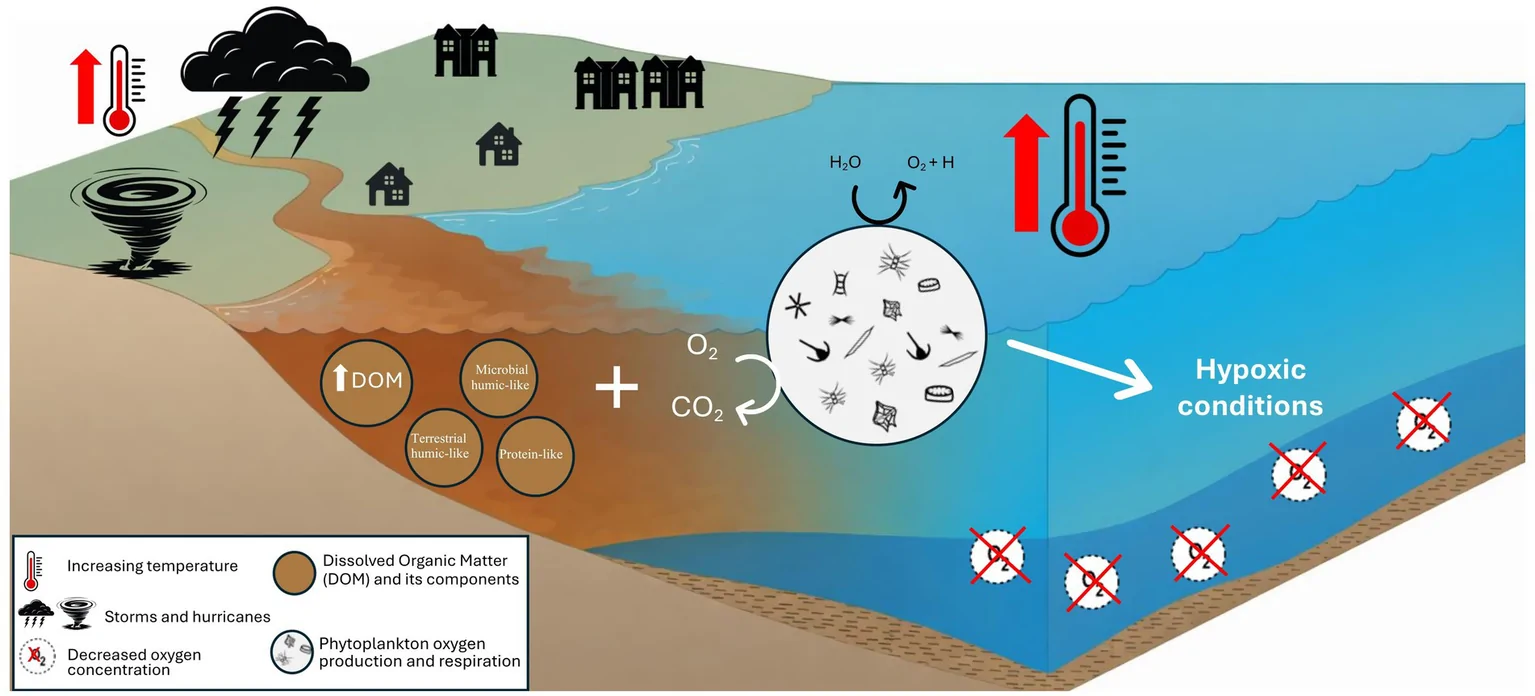
Conceptual figure illustrating the combined effects of warming and intensifying hurricanes on coastal biogeochemistry and oxygen dynamics in a subtropical system such as the Gulf of Mexico. Rising temperatures increase the frequency and intensity of tropical storms and hurricanes, enhancing rainfall, river discharge, and terrestrial runoff. These processes deliver elevated loads of dissolved organic matter (DOM) to coastal waters, including terrestrial humic-like, microbial humic-like, and protein-like components. Simultaneously, warming of surface water increases water-column stratification, reducing vertical mixing and oxygen ventilation of deeper waters, while higher temperatures decrease oxygen solubility. Under warmer conditions and increased DOM availability, respiratory processes are favored, while primary production is reduced, shifting the system toward net heterotrophy. The combined effects of enhanced respiration, reduced oxygen, and lower oxygen solubility promote oxygen depletion and increase the likelihood of hypoxic conditions. Icons were adapted from the Integration and Application Network (IAN), University of Maryland Center for Environmental Science (UMCES), and Adobe Illustrator. The brownish water effect was added using AI-assisted editing.

Taking advantage of the series of hurricanes that occurred in 2023 and 2024 along the WFS, this study examined how increased temperature and humic acid enrichment, both under *in situ* post-hurricane conditions and through controlled experimental manipulations, affect the metabolic rates of the phytoplankton community. We hypothesized that increasing temperature, combined with hurricane-driven inputs of humic acids, would reduce primary production while stimulating respiration processes and DOM degradation. We also hypothesized that light dependent respiration, exclusively due to algal respiratory processes and measurable with ^18^O techniques, would be significant compared to microbial community respiration. Together, these processes should shift the metabolic balance toward greater net heterotrophy, thereby increasing the likelihood of hypoxia development in WFS waters. To assess these effects, gross primary production (GPP), net primary production (NPP), gross respiration (i.e., the sum of algal light-dependent metabolic processes, GR), and dark community respiration (CR) rates of dissolved O_2_ were measured. Changes in the chemical character of fluorescent DOM (FDOM) as a function as temperature were also measured. These findings provide new insight into how climate-related stressors may interact to influence the balance between autotrophic and heterotrophic processes in coastal phytoplankton communities with important links to, and implications for, the development of hypoxia conditions.

## 2 Materials and methods

### 2.1 Overview and sampling

All sampling was opportunistic, with water collection and experiments as soon after various hurricanes as practical and at sites that could be conveniently accessed from land or from available boats. Different types of experiments or measurements were undertaken for all post hurricane sampling in 2024, depending on date of sampling (Table 1). Since sampling was conducted opportunistically as part of a rapid-response effort, the environmental parameters collected varied among sampling events depending on field conditions and logistical constraints (Table 1).

**Table 1:** Summary of sampling efforts following Hurricanes Idalia, Debby, Helene, and Milton, including the stations where water was collected and the experiments conducted for each event.

Hurricane	Station sites	Experiments
Idalia (Category 4; landfall at Big Bend, Florida)	Charlotte Harbor (CH)	Pre hurricane rates of gross respiration
Debby (Category 1; landfall at Steinhatchee, Florida)	Tampa Bay (TB), Siesta Key (SK), Caloosahatchee River (CR) and Charlotte Harbor (CH)	Primary production and gross respiration rates vs. temperature Dark and light respiration rates
Helene (Category 4; landfall near Perry, Florida)	New Pass (NP) and Siesta Key (SK)	Primary production and gross respiration rates vs. temperature FDOM transformation vs. temperature Dark and light respiration rates
Milton (Category 5; landfall near Siesta Key, Florida)	New Pass (NP) and Siesta Key (SK)	Primary production and gross respiration rates + humic acid enrichment vs. temperature FDOM transformation + humic acid enrichment vs. temperature Dark and light respiration rates Dark respiration vs. temperature

Site locations and hurricane tracks for the 2024 hurricanes are shown in Figure 2.

All samples used in experiments were collected from rivers discharging onto the WFS, or from coastal WFS waters in August and September 2023 (5 days before and 7 days after Hurricane Idalia) and between August and November 2024 after Hurricanes Debby (August 8, 5 days post storm), Helene (October 1, 6 days post storm), and Milton (November 20–23, 6 weeks post storm; Figure 2). Sampling efforts in August 2023, and also following Hurricanes Debby and Helene, were conducted on a single day, whereas sampling after Hurricane Milton extended across 3 days.

**Figure 2:**
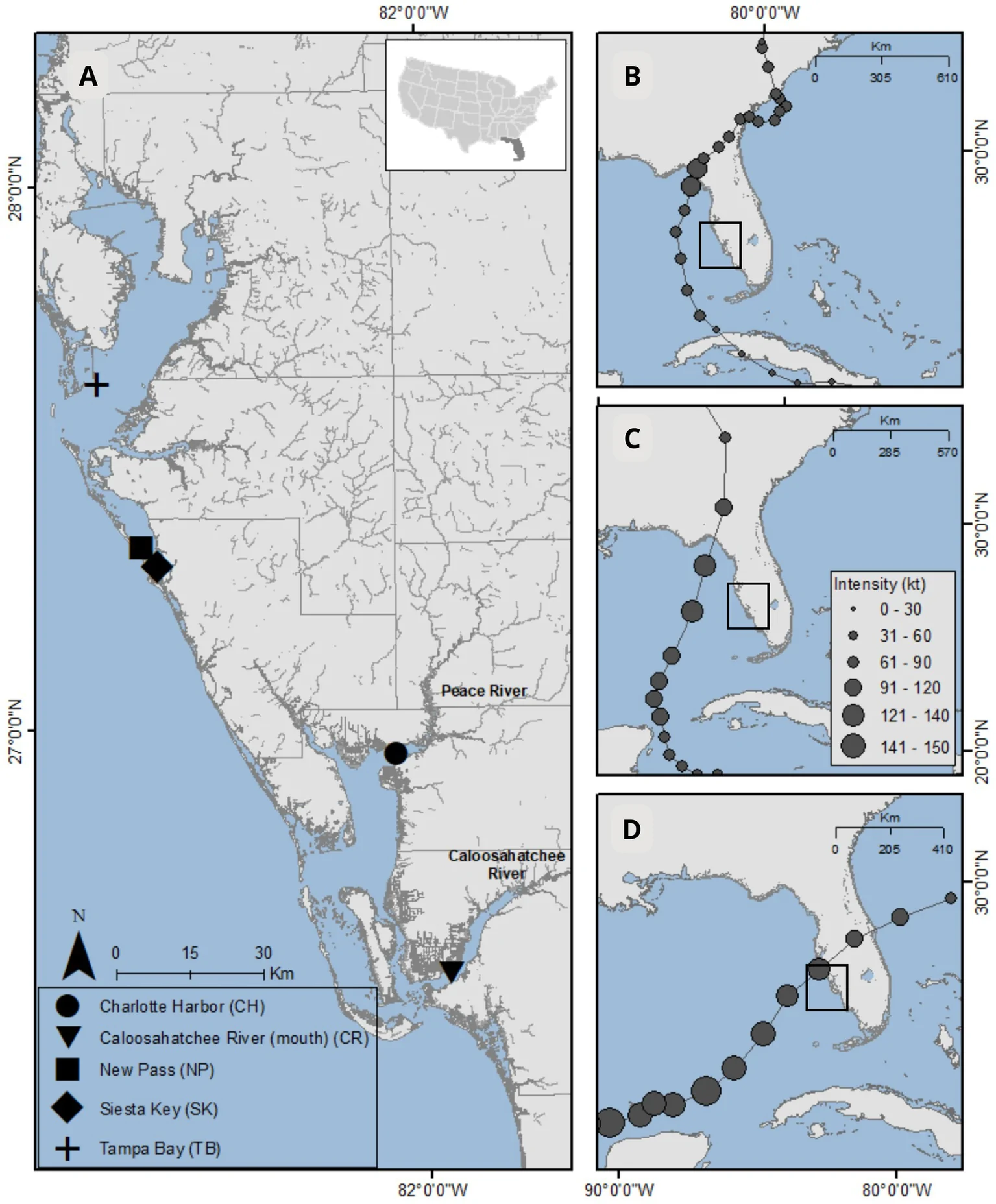
**(A)** Map of sampling sites on the West Florida Shelf: Charlotte Harbor (CH), Caloosahatchee River (mouth; CR), New Pass (NP), Siesta Key (SK) and Tampa Bay (TB). Panels **(B–D)** show the tracks of Hurricane Debby **(B)**, Helene **(C)** and Milton **(D)** along the West Florida coast. Hurricane intensity data were obtained from the RAMMB Tropical Cyclone Realtime database hosted by the Cooperative Institute for Research in the Atmosphere (CIRA), Colorado State University. The outlined boxes on panels **(B–D)** define the region from panel **(A)**.

Pre- and post- Hurricane Idalia sampling involved collections of ambient nutrient conditions, and measurements of the light dependency of GR in Charlotte Harbor. The pre-hurricane measurements, collected as part of routine sampling and not in anticipation of a pending hurricane, also serve as a comparative non-impacted summer condition, against which the rate measurements following Hurricane Debby can be compared.

Experiments conducted in the days to weeks following the different hurricanes that occurred in 2024, were designed to assess the effects of natural organic matter loads and temperature on primary production and respiration. Three types of experiments were performed, although not all experiments were performed following each event. First, rates of productivity and respiration across a range of temperatures were performed on discrete samples from nearshore sites and from offshore sites when boat collection permitted. Second, some samples were also inoculated with addition of humic material extracted from water collected from the humic-rich Shark River Slough in southwest Florida (details below). Productivity and respiration rates were measured across ambient and manipulated temperatures (+ 3–5 °C) in the light to reflect light-dependent respiration (i.e., GR) in all sampling efforts and dark respiration were made across a temperature gradient in one sampling effort. Finally, in a subset of samples, the transformations of FDOM were assessed in temperature-manipulated incubations (Table 1).

### 2.2 Ambient parameters, nutrients, and chlorophyll-*a* analyses

In the field, water was collected with a Niskin bottle lowered to ~5 cm below the water surface then transferred to clean (10% HCl, followed by three deionized water rinses) bottles or carboys. All water collected was initially screened at the time of sample collection through a 100 μm nylon mesh to separate the smaller fractions of phytoplankton from zooplankton. Measurements of temperature and salinity were taken on each sample on site using a ProQuatro and a YSI ProSolo. Samples were returned to the laboratory within 2 h of sampling and immediately filtered through precombusted GF/F filters (450 °C, 2 h).

For samples pre- and post- Hurricane Idalia and post- Hurricane Helene, determinations of NO_3 + 2_ (hereafter termed NO_3_^−^), PO_4_^3−^, NH_4_^+^ and Chlorophyll-*a* (Chl-*a*) were undertaken. Filtrates and filters were stored frozen. All nutrient analyses were carried out at the Mote Marine Laboratory using a continuous segmented flow analyzer (Auto-analyzer 3 SEAL) according to Dixon et al. (2014). Concentrations of Chl-*a* were analyzed using high-performance liquid chromatography (HPLC, Agilent HPL) at the Horn Point Analytical Services Laboratory according to Van Heukelem and Thomas (2001). In addition, dissolved organic carbon (DOC) concentrations were measured for the same samples according to Dickson et al. (2007), and chromophoric dissolved organic matter (CDOM), was quantified as absorbance at 280 nm. Cell abundance and classification were performed on all raw water samples preserved with Lugol’s solution (5%) in a Sedgewick-Rafter chamber (1 mL) using a Zeiss Axio Vert.A1 inverted microscope.

### 2.3 Humic acid extraction

All humic material used in enrichment studies was originally collected and isolated from the Shark River Slough, Florida, several years prior to this study, and stored frozen until analysis. The water samples for humic extractions were collected in precleaned carboys (10% HCl followed by three deionized water rinses). Water was returned to the lab and filtered through precombusted GF/F filters within 6 h of collection and stored at 4 °C in darkness until further extraction. Humic acids were extracted according to a modification of the method of Leenheer (1981) using XAD-8 resin (Sigma Chemical) and Duolite A-7 anion exchange resin, as previously described in Heil (2005). The XAD-8 resin was cleaned (sequential 100% washes of diethyl ether, acetonitrile, methanol, and ether), slurried with distilled water, and packed into glass columns. The columns were subsequently cleaned and prepared as per Heil (2005). Just before extraction, the filtered water sample was acidified to pH 2.0 with 6.0 N HCl. Then, approximately 40 liters of the sample was passed through the XAD-8 column, which was subsequently rinsed with 100 mL of distilled water, followed by elution with 0.1 N NaOH. The resulting eluant was acidified with 6.0 N HCl (Liao et al., 1982; Bowles et al., 1989), then centrifuged (3,000 g, 20 min). The resulting pellet, representing the humic material, was washed with distilled water, recentrifuged, and dissolved in 0.1 N NaOH, passed through a cation exchange column, reacidified, and recentrifuged again. The dried humic fraction was dissolved in 50 mL of milli-Q water to make a humic stock solution with a concentration of 1 mg L^−1^ then filtered (0.2 μm Nuclepore filter) and stored in darkness at 4 °C.

### 2.4 Primary production and respiration rate measurements

After return to the laboratory, portions of the screened water samples (30–60 mL) were enriched with ~1% of H_2_^18^O (Marshall Isotopes Ltd.) and dispensed without headspace into 12–15, 3 mL screw-capped vials sealed with Viton septa. The application of the H_2_^18^O tracer measures primary production simultaneously with algal respiratory processes; derived rates do not include respiratory processes associated with bacteria or other microbes within a natural sample. This is due to the fact that the source water used in photosynthesis is enriched with the heavy isotope and as algal productivity occurs, water is split and the produced, now isotopically enriched O_2_, can be subsequently respired by the cell via various process in the chloroplast as well as in the mitochondria (Section 2.5).

To assess algal GR (i.e., the sum of light-dependent metabolic processes) in the pre-Idalia experiments, vials were incubated in Photosynthetrons (MacIntyre et al., 1996), which are incubation chambers with light ranging from 0 to 800 μmole photons m^−2^ s^−2^. For all subsequent experiments, incubations were conducted in chambers with constant light (~130 μmol photons m^−2^ s^−1^). These were custom made, hereafter called “Coronations” (Bay Instruments LLC; Sobrinho et al., 2026) and designed to hold 12 individual vials each. Then, the Coronations were placed in three different constant temperature incubators for up to 8 h (Figure 3). Depending on sample or experiment and water quantity available, vials represented single, replicate or triplicate treatments (Table 1). Rates of GR coincident with rates of GPP, NPP were calculated for all treatments as described further below. The constant temperature treatments included near-ambient temperature, ambient temperature +3 °C, and ambient temperature +6–8 °C. Before incubation of all experiments, triplicate vials were immediately fixed with mercuric chloride (HgCl_2_, ~1% v/v), representing a time zero value. At the end of the incubation period, samples were removed from the Coronations, fixed with HgCl_2_ (~1% v/v), placed completely underwater in jars at room temperature until analysis (within days) to minimize air contamination. Subsequent analysis was performed by Membrane Inlet Mass Spectrometry (MIMS) (Bay Instruments LLC, Easton, Maryland) (Sobrinho et al., 2026). Rates of GPP, NPP, and GR were normalized by the Chl-*a* concentration of each sample.

**Figure 3:**
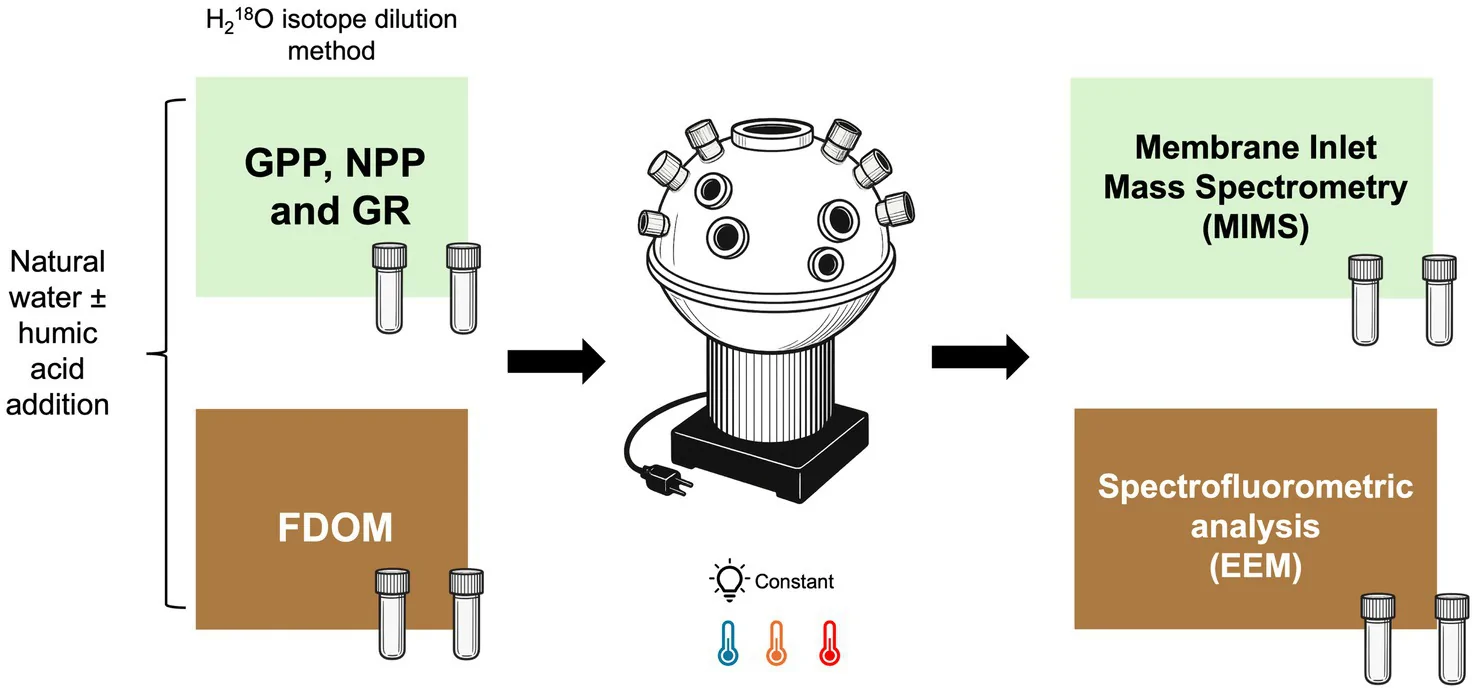
Schematic overview of the incubation setup used to quantify primary production (GPP, NPP) and gross respiration (GR), as well as changes in dissolved organic matter (FDOM) composition. Water samples were dispensed into 3-mL gas-tight vials and incubated for up to 8 h in custom-built incubators (“Coronations”), each maintained at constant light and at three temperature treatments (ambient, ambient + 3 °C, and ambient + 6–8 °C). At the end of the incubation, vials enriched with ~1% H_2_^18^O were fixed and analyzed by Membrane Inlet Mass Spectrometry (MIMS) to quantify metabolic rates. For select sites/dates, approximately 1 mL from each vial was transferred into an acid washed (10% HCl) and baked (232 °C, 2 h) scintillation vial and placed in the refrigerator (4 °C) in the dark for fluorescence excitation-emission matrix-parallel factor (EEM-PARAFAC) analysis of FDOM. ChatGPT was used to generate the Coronations icon.

An additional experiment was conducted post-Hurricane Milton, involving enrichment of samples with 6 mg L^−1^ of the previously extracted humic acids prior to enrichment with ~1% of H_2_^18^O. The amount of humic acid enrichment was based on prior measurements of colored DOM (CDOM) from samples from Charlotte Harbor (May and August 2023; E. Hall, unpublished data), which ranged from 1.3 to 12.9 mg L^−1^.

To measure CR as a function of water temperature, water samples with no isotope added were carefully dispensed in 7 mL vials and placed in a dark, custom-made temperature control block with cool water circulating at one end and warm water at another, yielding a gradient of five values ranging from −5 °C to +5 °C relative to ambient temperature. After incubation (6–8 h), vials were stored in water filled jars as described above and subsequently analyzed by MIMS as described below (Sobrinho et al., 2026).

In parallel with the measurements of GPP, NPP and GR in the Coronatron incubations for samples collected following Hurricanes Debby and Helene, a replicate set of vials with no isotope enrichments was incubated in the Coronations for subsequent analysis of FDOM. After incubation, vials were frozen for subsequent spectrofluorimetric analysis at the University of South Florida (Horiba Aqualog, Horiba Instruments Inc.) as described in detail below.

### 2.5 Calculations and statistical analyses

To determine the rates of GPP, NPP, and GR, the mass-to-charge ratios (m/z) of the relative abundance of dissolved ^16^O:^16^O (mass 32), ^18^O:^18^O (mass 34), and Ar (Argon, mass 40) in the samples were measured by membrane inlet mass spectrometry (MIMS; Sobrinho et al., 2026). Data analysis was by the software Quickdata (Bay Instruments, LLC) according to Kana (1990), Kana et al. (1994), and Ferrón et al. (2016). Rates of GPP were calculated based on the change in the isotope ratio of dissolved O_2_ over the incubation time (mass 34/40) (Equation 1).


$$\mathrm{GPP}=(\frac{\left(\Delta [\frac{34}{40}]\times [\mathrm{Ar}]\right)}{2*(\;18^{O}(H_{2}O)+0.002)*t})$$ (1)

where Δ34/40 , represents the change in the isotope relative to Ar ratio over time; [Ar] is the solubility of Ar at the incubation temperature; ^18^O(H_2_^18^O) is the isotope ratio of the incubation water, which is calculated based on the amount of ^18^O(H_2_^18^O) added in the total volume of the incubation flask and the percent enrichment (98–99%) of stock ^18^O water; 0.002 represents the natural abundance of ^18^O; and *t* represents the incubation time. This equation provides ^18^O-GPP in μmol O_2_ h^−1^.

Rates of NPP were determined from the net change in O_2_/Ar (mass 32/40) (Equation 2).


$$\mathrm{NPP}=\frac{\Delta 32/40\times [\mathrm{Ar}]}{t}$$ (2)

where Δ32/40 represents the change in the O_2_/Ar over time, [Ar] is the solubility of Ar at a certain temperature, and *t* represents the incubation time. This equation provides ^18^O-NPP in μmol O_2_ h^−1^. Then, gross O_2_ uptake (GR) was determined from the difference between GPP and NPP. Finally, GPP, NPP, and GR ratios obtained were normalized by the Chl-*a* concentration of each sample, providing μmol O_2_ (μg Chl-*a*) ^−1^ h^−1^.

Rates of CR were determined by the difference in O_2_ concentration measured in the dark before and after incubation. Concentrations were measured using MIMS.

### 2.6 Data and statistical analyses of productivity and respiration

The normality of data was checked with the Shapiro-Wilks test, and the homogeneity of variance was assessed. All statistical analyses were performed using the R language (R Core Team, 2021). To test the relationship between CR and temperatures, linear models were used.

### 2.7 Dissolved organic matter analysis

For samples collected after Hurricanes Helene and Milton, the effects of temperature on the composition of fluorescent DOM (FDOM) were determined using fluorescence excitation-emission matrix-parallel factor analysis (EEM-PARAFAC; Stedmon and Bro (2008)). This technique was unavailable for the prior sampling efforts. This is a nondestructive technique that detects fluorophores at low concentrations in a three-dimensional landscape. This landscape, or EEM, is then mathematically deconvoluted via PARAFAC to identify fluorescent compounds such as humic acids and protein-like compounds (Muni-Morgan et al., 2024). Two indices, the biological index (BIX) and humification index (HIX) were calculated using the EEM absorbance and fluorescence spectra to resolve the degradation state, origin, and reactivity of DOM using the staRdom package in ‘Rstudio’ (version 4.2.2). The BIX is calculated using the ratio of fluorescence intensity of emissions 380/430 nm, obtained at an excitation wavelength of 310 nm (Huguet et al., 2009). Values > 1 are indicative of recent autochthonous production. The HIX is calculated by dividing the sum of emission intensities in the 435–480 nm region by the sum of total fluorescence intensities between the 300–345 nm and 435–480 nm regions, where the value of 1 is the highest degree of humification (Ohno, 2002). High values of HIX are associated with DOM that is higher in molecular weight, more aromatic, and of terrestrial origin.

EEM scans were performed on all samples (*n* = 60) using a Horiba Aqualog (Horiba Instruments Inc). Prior to analysis, all samples were brought to a final volume of 3 mL using Milli-Q water and stabilized to room temperature prior to fluorescence analysis. Samples were analyzed in an acid-soaked (10% HCl; 24 h) 1 cm quartz cuvette, which was rinsed 20 times with Milli-Q water before being scanned. Before sample analysis, validation checks for water Raman peak signal-to-noise and emission calibration were performed, as well as standardization of fluorescence intensity in quinine sulfate units (QSU) using a 1 ppm standard of quinine sulfate solution scanned at an excitation wavelength of 347.5 nm. Excitation wavelengths were measured from 239 to 800 nm (3 nm increments), and emission wavelengths were measured from 248 to 827 nm (5 nm increments).

Fluorescence EEM spectral correction was performed through subtraction of a sample blank of Milli-Q water to remove Raman scatter. The EEMs were corrected for inner filter effect (IFE), 1st and 2nd order Rayleigh scattering and normalized to the Raman peak of the Milli-Q water (Aqualog Software v 3.6) to enable a direct comparison of relative fluorescence between all sample EEMs. A single PARAFAC model was created in PLS Toolbox v 9.2 (Eigenvector Research Inc., Manson, WA, USA) in MATLAB (R2023a) to determine the underlying fluorophores in all samples using methods outlined in Stedmon and Bro (2008). The resultant model explained 99.1% of the data, which was validated using split half analysis (86.1), the core consistency diagnostic (61), and visual examination of residuals. The PARAFAC model was exported to the online spectral database OpenFluor to match underlying components to the existing literature using a Tucker congruence of 0.95 on the uploaded emission and excitation spectra (Murphy et al., 2014). The FDOM peaks were further compared visually to the commonly described Coble peaks (Coble, 1996).

The PARAFAC model components were identified as terrestrial humic-like (C1), microbial humic-like (C2), and protein-like (C3). The C1 component has two excitation maxima at <250 and 370 nm, and one emission maximum at 480 nm. It is defined by Coble (1996) as humic-like peak C. It is ubiquitous and has been observed in both terrestrial and marine DOM with minor differences in peak positions (Coble, 1996; Stedmon and Markager, 2005). This component is indicative of allochthonous, or terrestrially derived humics. The C2 component matches the spectral characteristics of Coble peak M and is considered microbially produced or autochthonous humics of recent biological origin (Coble, 1996). It has two excitation maxima at <250 and 300 nm, and one emission maximum at 400 nm. Lastly, the C3 component is defined as Coble peak T, resembling the spectra of tryptophan (Coble, 1996). It has an excitation/emission maximum at 270/310 nm. Protein-like DOM is considered highly labile and can originate via biological production or cell lysis. Tryptophan is also used a marker for anthropogenic DOM originating from wastewater (Harris et al., 2024).

## 3 Results

### 3.1 Ambient condition and phytoplankton community

Before and after Hurricane Idalia (August 2023), samples were collected in Charlotte Harbor (CH). The environmental conditions changed substantially following the hurricane. Although water temperature remained similar before and after the storm (29.6 and 28.8 °C, respectively), salinity declined from 23.7 to 16.0. Nitrogen concentrations increased markedly, with NH_4_^+^ rising from 1.63 to 3.41 μM, and NO_3_^−^ from <0.07 to 2.56 μM (Supplementary Table 1). Concentrations of DOC also increased from 686 to 1,080 μM, while CDOM nearly doubled, increasing from 39.5 to 88.5 m^−1^ (Supplementary Table 1).

Sampling following Hurricane Debby (August 2024) involved multiple stations: Tampa Bay (TB), Siesta Key (SK), Caloosahatchee River (CR) and Charlotte Harbor (CH). Following the hurricane, water temperatures were similar across all stations (28 °C). Chlorophyll (Chl-*a*) concentrations varied considerably across the stations: concentrations were 1.84 μg L^−1^ at TB, 3.59 μg L^−1^ at SK, 2.32 μg L^−1^ at CR, and 4.31 μg L^−1^ at CH (Supplementary Table 1). Phytoplankton community composition also varied considerably (Supplementary Table 2). Station TB exhibited the highest total cell abundance and was dominated by diatoms (~1.28 × 10^7^ cells L^−1^), with relatively small contributions from other taxa (~2.4 × 10^5^ cells L^−1^). At SK, total abundance was lower, with cyanobacteria (~9.6 × 10^5^ cells L^−1^) representing the dominant group, followed by diatoms (~6.6 × 10^5^ cells L^−1^). Station CR had the lowest overall cell abundance (<2 × 10^5^ cells L^−1^), with diatoms comprising most of the community. Cell abundance was higher at CH relative to CR, where cyanobacteria (~6.6 × 10^5^ cells L^−1^) and diatoms (~2.2 × 10^5^ cells L^−1^) represented the majority of the phytoplankton community (Supplementary Table 2).

After Hurricane Helene (October 2024), when sampling was limited to sites New Pass (NP) and SK, water temperature remained similar between stations (28 °C), while Chl-*a* concentrations reached 10.09 and 8.55 μg L^−1^ at NP and SK, respectively (Supplementary Table 1). Phytoplankton community composition differed between the two stations (Supplementary Table 2). At NP, the community was dominated by diatoms (4.60 × 10^5^ cells L^−1^), with smaller contributions from dinoflagellates (4 × 10^4^ cells L^−1^) and other taxa (~3 × 10^4^ cells L^−1^). In contrast, SK exhibited substantially higher cell abundance, largely dominated by cyanobacteria (~1.13 × 10^7^ cells L^−1^). Microscopic examination showed that many of these cells consisted of small coccoid forms embedded within mucilaginous aggregates that did not appear physiologically healthy. These cells were identified as *Microcystis* sp. colonies, most likely transported by runoff from Phillippi Creek, which discharges near the SK station during rainfall events. Although the colonies observed at SK were likely non-viable, their morphology remained readily identifiable. Diatoms (~5.40 × 10^5^ cells L^−1^), dinoflagellates (~8 × 10^4^ cells L^−1^), and other taxa (~1.10 × 10^5^ cells L^−1^) contributed comparatively little to total abundance. The overwhelming dominance of cyanobacteria at SK indicates a stronger influence of freshwater inputs and nutrient enrichment than at NP.

Post Hurricane Milton, sampling again focused on sites NP and SK and environmental conditions were relatively stable throughout the three sampling days over which sampling occurred. Water temperature was cooler than the earlier sampling periods, ranging from 19 to 23 °C, while salinity remained moderate at both NP and SK (31–33). Nutrient concentrations were generally low at both stations, with NH_4_^+^ (3.7–3.9 μM) representing the dominant inorganic N form. In contrast, NO_3_^−^ and PO_4_^3−^ concentrations remained below 1 μM throughout the sampling period (Supplementary Table 1). Phytoplankton community composition was broadly similar among sampling days, although total cell abundance varied between stations and over time (Supplementary Table 2). On Day 1, SK exhibited the highest total cell abundance, dominated by diatoms (~4 × 10^6^ cells L^−1^), with *Skeletonema costatum* accounting for approximately 80% of the community. In contrast, NP had lower total abundance (8 × 10^5^ cells L^−1^) but a proportionally greater contribution of *K. brevis* (~4 × 10^5^ cells L^−1^). At NP, total cell abundance declined markedly on Day 2, although diatoms remained the dominant group. By Day 3, total cell abundance increased to approximately 3.6 × 10^6^ cells L^−1^, returning to levels comparable to those observed on Day 1 and continuing to be dominated by diatoms. *K. brevis* was consistently present at moderate abundance throughout the three sampling days (6 × 10^4^ – 4 × 10^5^ cells L^−1^).

### 3.2 Primary production and gross respiration rates as a function of temperature

Strong differences were seen across sampling periods and stations and treatments, but there were several general patterns. Rates of GPP generally declined with temperature, while rates of GR increased with temperature, typically leading to rates of NPP that were very low, in some cases negative. This increased heterotrophy was due to algal respiration, GR, which in at least one sampling period, was higher than rates of dark respiration, CR.

Following Hurricane Idalia, light-dependent gross respiration (GR) averaged 0.13 μmol O_2_ (μg Chl-*a*)^−1^ h^−1^ (Supplementary Figure 1). Rates increased as light first increased to 60 μmol photons m*^−2^* s^−1^, then progressively decreased with further light availability.

Following Hurricane Debby (August 2024), the sampled stations had a pronounced gradient in CDOM concentrations as evidenced from the discoloration of water, especially at the more inner-shelf stations, particularly at sites CR and CH (Figure 4). At ambient temperature, rates of GPP as well as GR were highest at the TB site, and decreased progressively to SK, CR and CH (Figure 5). Rates of GPP also declined across all stations as temperature increased (Figure 5). Rates of GPP decreased from 0.31–1.16 μmol O_2_ (μg Chl-*a*)^−1^ h^−1^ at 28 °C to 0.21–0.79 μmol O_2_ (μg Chl-*a*)^−1^ h^−1^ at 33 °C, representing reductions of up to 32%. The NPP rates followed a similar trend, decreasing from −0.006 to 0.663 μmol O_2_ (μg Chl-*a*)^−1^ h^−1^at^1^ at 28 °C to −0.536 to −0.073 μmol O_2_ (μg Chl-*a*)^−1^ h^−1^ at 33 °C. At the highest temperature, NPP was negative at all stations, as algal respiration rates exceeded those of gross photosynthesis. Rates of GR increased from 0.23–0.53 to 0.45–0.90 μmol O_2_ (μg Chl-*a*)^−1^ h^−1^ with increasing temperature, corresponding to increases of up to 109%. These responses were strongest at the more colored stations (CR and CH), where baseline GPP was already lower and warming produced the largest stimulation of GR.

**Figure 4:**
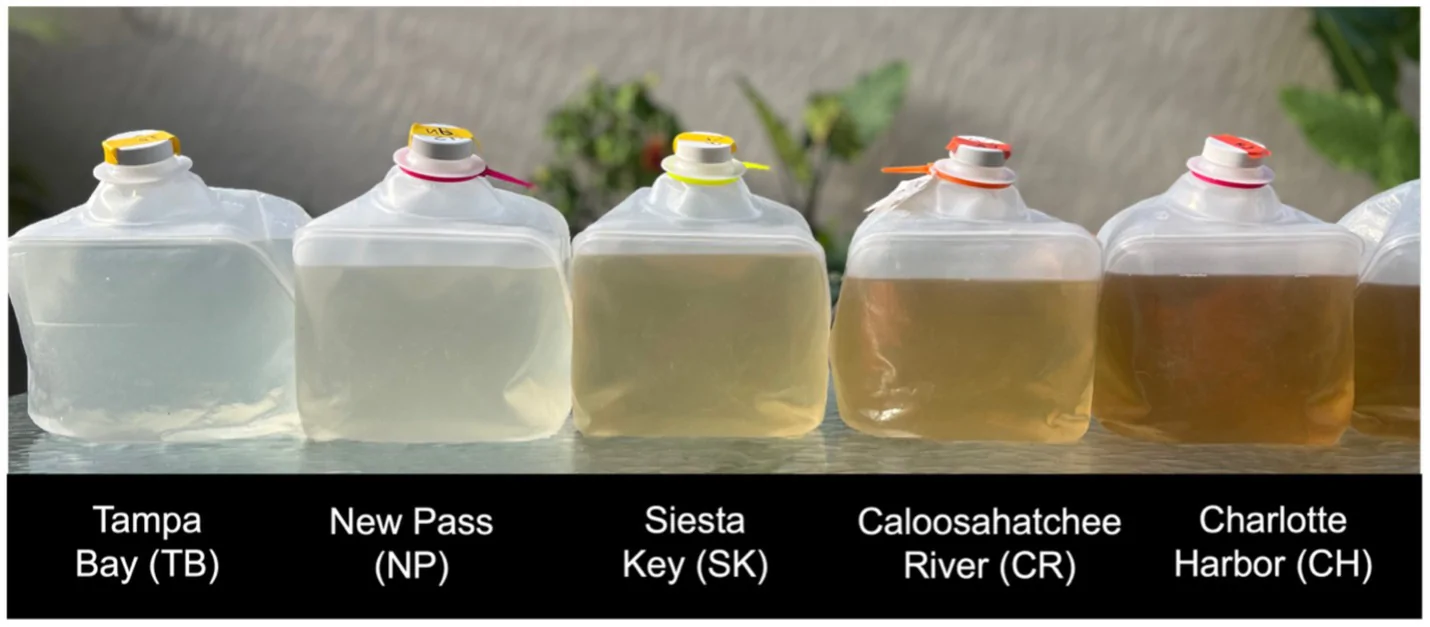
Visual gradient of water color across stations sampled from north to south: Tampa Bay (TB), New Pass (NP), Siesta Key (SK), Caloosahatchee River mouth (CR), and Charlotte Harbor (CH). The darkest waters (CH, CR) represent the most inland stations, where terrestrial influence and organic matter inputs are strongest.

**Figure 5:**
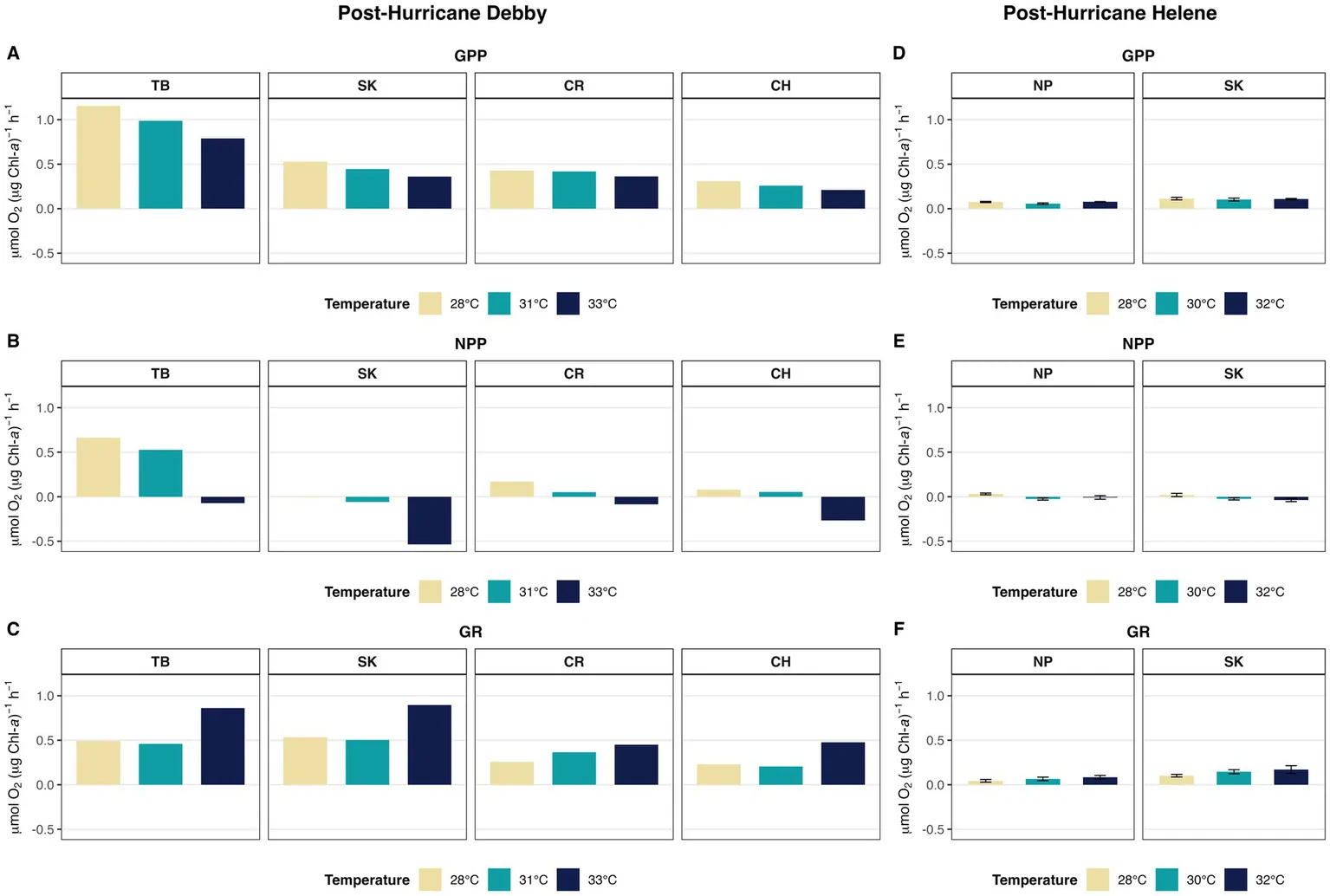
Gross primary production (GPP) **(A)**, net primary production (NPP) **(B)**, and gross respiration (GR) **(C)** (μmol O_2_ (μg Chl-a)^−1^ h^−1^) measured under three temperature treatments (28 °C, 31 °C and 33 °C) across four stations: Tampa Bay (TB), Siesta Key (SK), Caloosahatchee River mouth (CR) and Charlotte Harbor (CH) following Hurricane Debby (August 2024) and the same parameters (GPP **(D)**; NPP **(E)**; and GR **(F)**) for stations New Pass (NP) and Siesta Key (SK) following Hurricane Helene (October 2024). Note the different temperatures tested. All samples following Hurricane Helene were run in triplicate and error bars represent standard deviations.

After Hurricane Helene, some similar and some differing metabolic responses were observed at both the NP and SK sites compared to sites studied following Hurricane Debby (Figure 5). In this case, rates of GPP were low and changed little with increasing temperature, ranging from 0.05 to 0.07 μmol O_2_ (μg Chl-*a*)^−1^ h^−1^ at NP and from 0.10 to 0.11 μmol O_2_ (μg Chl-*a*)^−1^ h^−1^at SK (Figure 5D). Rates of NPP progressively declined from slightly positive values at 28 °C (0.03 μmol O_2_ (μg Chl-*a*)^−1^ h^−1^at NP and 0.01 μmol O_2_ (μg Chl-*a*)^−1^ h^−1^ at SK) to negative values at the highest temperature (−0.007 and −0.03 μmol O_2_ (μg Chl-*a*)^−1^ h^−1^ at NP and SK, respectively) (Figure 5E). Similar to Hurricane Debby, rates of GR increased progressively with warming, reaching its highest value (0.17 μmol O_2_ (μg Chl-*a*)^−1^ h^−1^) at SK at 32 °C (Figure 5F) but rates were overall lower than those following Debby.

Post-Hurricane Milton, experimental results reflected the same overall metabolic response as seen in the previous sampling efforts, although the magnitude differed between stations (Figure 6). These experiments also explored the responses with additions of supplemental humic acid. At NP, GPP remained positive but declined from 0.14 μmol O_2_ (μg Chl-*a*)^−1^ h^−1^ at 24 °C to 0.01 μmol O_2_ (μg Chl-*a*)^−1^ h^−1^ at 30 °C. The NPP rates also decreased at the NP site, from 0.05 to −0.45 μmol O_2_ (μg Chl-*a*)^−1^ h^−1^, while GR increased from 0.09 to 0.46 μmol O_2_ (μg Chl-*a*)^−1^ h^−1^. The addition of humic acids produced only minor changes relative to the controls, with slightly lower GPP, low but positive NPP (0.03–0.09 μmol O_2_ (μg Chl-*a*)^−1^ h^−1^), and a small increase in GR with temperature. The response at SK was considerably stronger than at NP. Rates of GPP declined from positive values at 24 and 27 °C (0.05–0.08 μmol O_2_ (μg Chl-*a*)^−1^ h^−1^) to negative values at 30 °C (−0.17 μmol O_2_ (μg Chl-*a*)^−1^ h^−1^). Likewise, NPP rates became increasingly negative, decreasing from −0.31 to −1.61 μmol O_2_ (μg Chl-*a*)^−1^ h^−1^, while GR rates increased from 0.36 to 1.44 μmol O_2_ (μg Chl-*a*)^−1^ h^−1^. Similar to site NP, at site SK, additions of humic acids resulted in a slight increase in GPP and NPP and a modest reduction in GR relative to the controls. Under these treatments, GPP decreased from 0.26 μmol O_2_ (μg Chl-*a*)^−1^ h^−1^ at 24 °C to −0.11 μmol O_2_ (μg Chl-*a*)^−1^ h^−1^at 30 °C, NPP declined from 0.02 to −1.29 μmol O_2_ (μg Chl-*a*)^−1^ h^−1^, and GR reached 1.17 μmol O_2_ (μg Chl-*a*)^−1^ h^−1^ at 30 °C.

**Figure 6:**
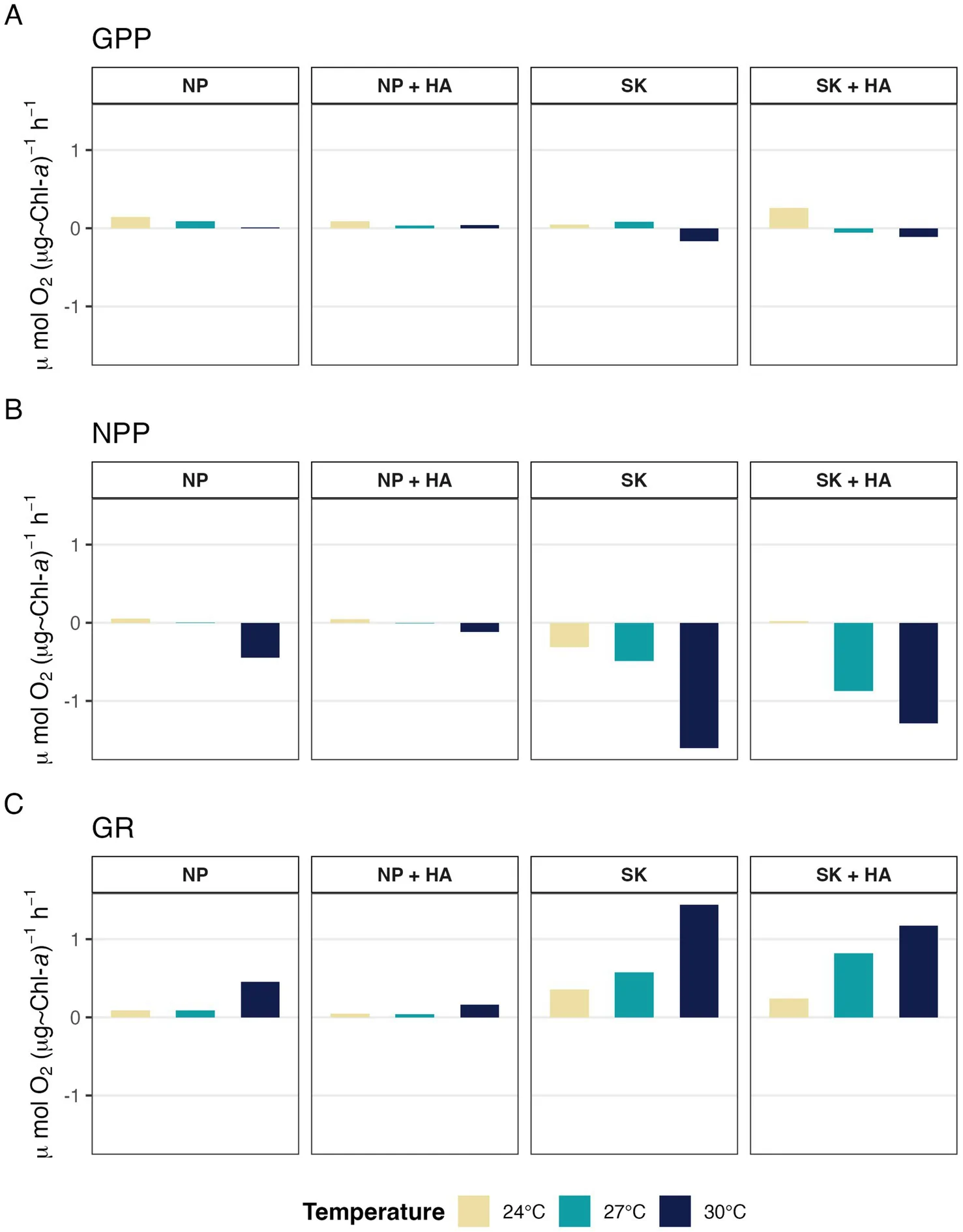
Gross primary production (GPP) **(A)**, net primary production (NPP) **(B)**, and gross respiration (GR) **(C)** at New Pass (NP) and Siesta Key (SK) with and without humic acid (HA) enrichment across three temperature treatments (24 °C, 27 °C, 30 °C) following Hurricane Milton.

### 3.3 Dark vs. light respiration rates

Comparisons of light and dark respiration (GR vs. CR) at ambient temperatures showed that GR rates, attributed solely to algal respiration, were of the same order of magnitude, if not higher, than CR, depending on sampling period or station (Figure 7). Following Hurricane Debby, GR and CR exhibited contrasting patterns among stations at the ambient temperature (28 °C) (Figure 7A). At site TB, the GR rate was over an order of magnitude higher than the rate of CR, 0.49 and 0.04 μmol O_2_ (μg Chl-a)^−1^ h^−1^, respectively. A similar pattern occurred in SK, where the GR rate (0.53 μmol O_2_ (μg Chl-a)^−1^ h^−1^) exceeded the CR rate (0.34 μmol O_2_ (μg Chl-a)^−1^ h^−1^) by 55%, indicating strong light-driven respiration in these clearer coastal waters. In contrast, the more colored waters, CR and CH, exhibited CR rates that were slightly higher than rates of GR. It should be noted that due to sample water availability, only single measurements were made of these rates during this sampling period. After Hurricane Helene, CR rates consistently exceeded GR rates, with the largest difference observed at SK (Figure 7B). Post-Hurricane Milton, CR rates also exceeded those of GR (Figure 7C).

**Figure 7:**
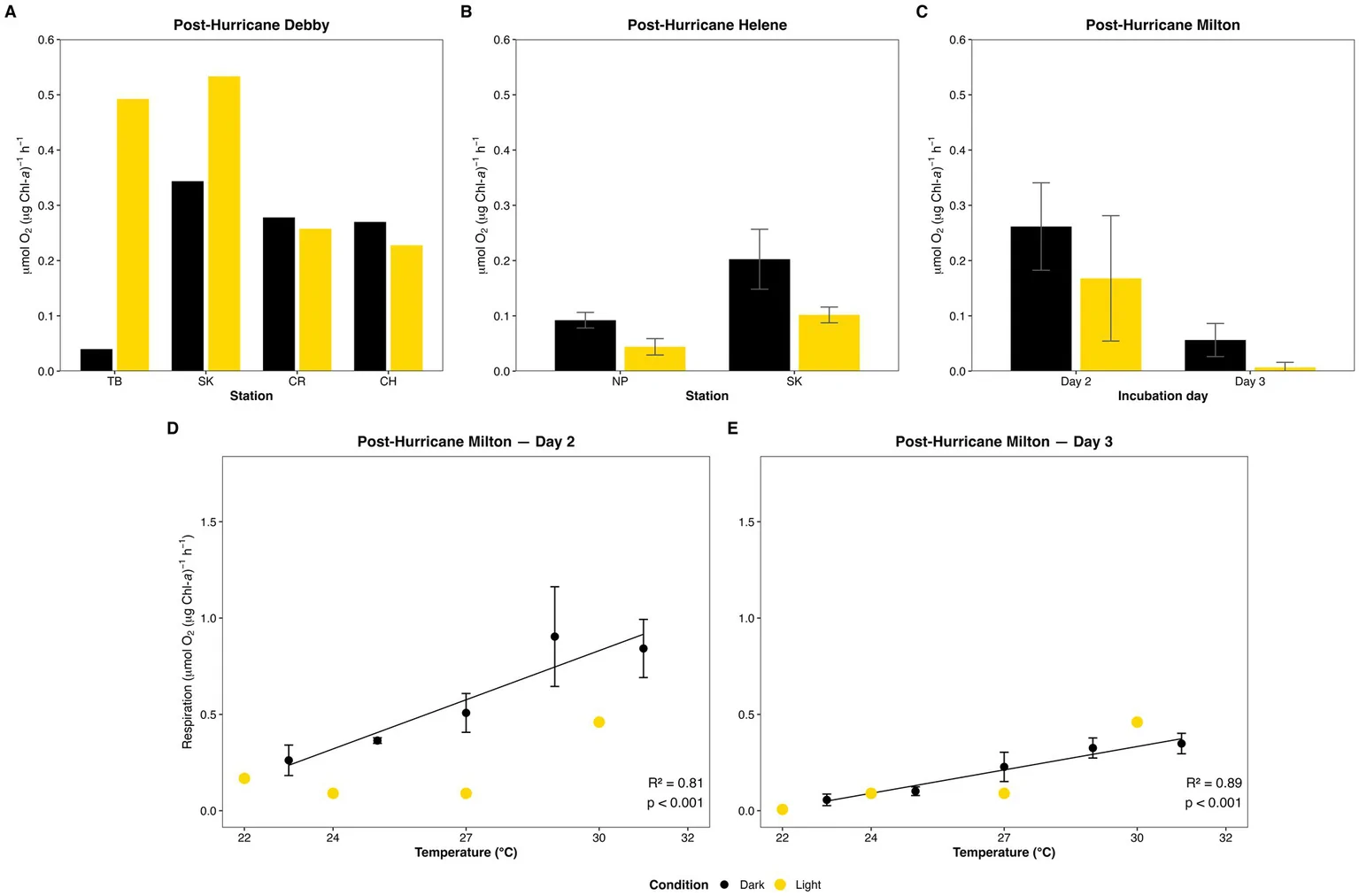
Comparison of dark respiration (CR; dark bars) and light-dependent gross respiration (GR; yellow bars) rates measured after three hurricane events at ambient temperatures. **(A)** Post-Hurricane Debby rates were measured at 28 °C across four stations: Tampa Bay (TB), Siesta Key (SK), the Caloosahatchee River mouth (CR), and Charlotte Harbor (CH). **(B)** Post-Hurricane Helene rates were measured at 28 °C at New Pass (NP) and Siesta Key (SK). **(C)** Post-Hurricane Milton mean respiration rates measured at ambient temperatures (23–24 °C) at NP on Days 2 and 3. For Panel A, only single measurements were made after Hurricane Debby, while triplicate measurements were made for each rate or the other dates (bars represent the mean ± (*n* = 3) standard deviation). **(D,E)** Rates of CR (μmol O_2_ (μg Chl-a)^−1^ h^−1^; black points) across a temperature gradient (23 to 33 °C) in NP on Days 2 and 3 after Hurricane Milton. Yellow points represent the mean light-dependent GR measured at 22, 24, 27 and 30 °C22 °C on the corresponding sampling day. Error bars represent the mean ± standard deviation (*n* = 3). The solid black line indicates the linear regression fitted using only the dark respiration measurements.

Following Hurricane Milton, CR increased linearly with temperature at NP during both Day 2 and Day 3 of the experiment (R^2^ = 0.81 and 0.89, respectively, and *p*-values < 0.001; Figures 7D,E). Rates of CR increased from approximately 0.20 and 0.01 μmol O_2_ (μg Chl-*a*)^−1^ h^−1^ at 24 °C to 0.55 and 0.59 μmol O_2_ (μg Chl-*a*)^−1^ h^−1^ at 32 °C on Days 2 and 3, respectively, representing more than a 10-fold increase across the experimental temperature range. Although fewer temperatures were tested for GR compared to CR, when the GR rates are compared across the temperature gradient, rates were lower for Day 2, but across the temperature gradient, rates were comparable on Day 3 even though the points measured at ambient temperature (27.5-28 °C) showed differences (compare Figure 7E with Figure 7C).

### 3.4 FDOM transformations

Post-Hurricane Helene, warming resulted in shifts in relative fluorescence of all FDOM components in both NP and SK (Figures 8A–C). Mean initial BIX values at T0 were lower at site SK (0.79) vs. NP (0.91) (Table 2), suggesting a smaller contribution of recently produced, microbially derived FDOM at SK relative to NP. Values of FDOM reflected more humified organic matter at site SK with mean initial HIX values of 0.81 at T0 vs. 0.61 at the NP site. Consistent reductions in component C1 (Terrestrial humic-like) were observed at all temperatures at both sites SK and NP. Increases in C2 (Microbial humic-like) were only observed in NP samples vs. SK. Increases in C3 (Protein-like) were consistent with increasing temperature for NP and especially SK, along with increases in BIX. Conversely, the FDOM pool became less humified with increasing temperature as indicated by decreasing HIX values with increasing temperature at both sites.

**Figure 8:**
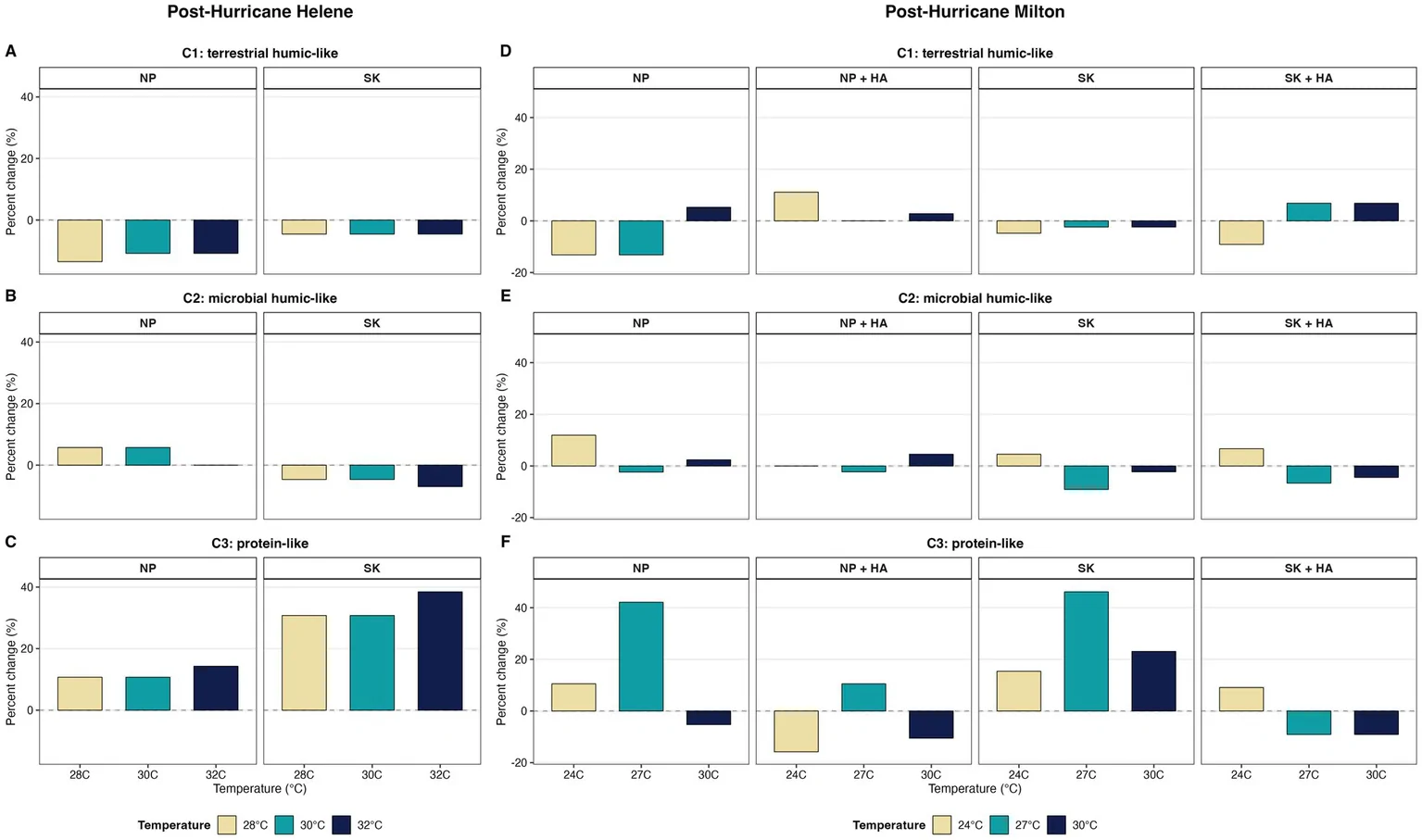
Panels **(A–C)** percent change in each FDOM component following Hurricane Helene (C1 = terrestrial humic-like (panel **A**), C2 = microbial humic-like (panel **B**), C3 = protein-like (panel **C**)) generated by the PARAFAC model for each site (New Pass, NP; Siesta Key, SK). Ambient temperature was 28 °C and tested temperatures included 28 °C, 30 °C, and 32 °C. Panels **(D–F)** Percent change in each FDOM component following Hurricane Milton for NP and SK. For each site a separate treatment also included additions of humic acid (denoted +HA). Ambient temperature was ~22 °C and the tested temperatures included 24 °C, 27 °C, and 30 °C.

**Table 2:** Mean biological index (BIX) and humification index (HIX) values ± standard error of the mean at sites SK and NP across temperature treatments following Hurricane Helene.

Treatment	Site	BIX	HIX
T0	SK	0.79 ± 0.01	0.81 ± 0.01
28 °C	SK	0.80 ± 0.02	0.79 ± 0.01
30 °C	SK	0.78 ± 0.01	0.76 ± 0.03
32 °C	SK	0.86 ± 0.01	0.73 ± 0.04
T0	NP	0.91 ± 0.03	0.63 ± 0.03
28 °C	NP	0.92 ± 0.02	0.61 ± 0.03
30 °C	NP	0.94 ± 0.01	0.60 ± 0.04
32 °C	NP	1.00 ± 0.05	0.61 ± 0.05

For all samples, *n* = 3.

Following Hurricane Milton, FDOM composition also responded to increasing temperature at both stations, although treatments amended with humic acids exhibited a more stable DOM pool than the unamended treatments (Figures 8D–F). In general, C1 (Terrestrial humic-like) and C2 (Microbial humic-like) were more stable than C3 (Protein-like) where a greater percent change in relative contribution was observed. The percentage of C1 declined with increasing temperature at NP and SK but increased with the additions of humic acids. Contributions of C2 remained relatively stable at 24 °C across all treatments but generally decreased with increasing temperature. Values of BIX at NP (0.98) were higher than those at SK (0.89) at T0, consistent with the previous experiment post Hurricane Helene (Table 3). However, BIX was higher at both NP and SK post Hurricane Milton vs. post Helene. Additions of humic acids increased the humification of the FDOM pool as indicated by greater HIX values at T0 in the humic acids amended vs. unamended treatments (Table 3). Reductions in HIX with increasing temperature were observed in the unamended treatments except for NP at 30 °C. Conversely, HIX values increased with the humic acids amended treatments but did not show a consistent trend with increasing temperature.

**Table 3:** Mean biological index (BIX) and humification index (HIX) values at sites New Pass (NP) and Siesta Key (SK) with and without humic acid (HA) enrichment across temperature treatments following Hurricane Milton.

Treatment	Site	BIX	HIX	*n*
T0	SK	0.89 ± 0.02	0.77 ± 0.00	3
24 °C	SK	0.90	0.76	1
27 °C	SK	0.88	0.73	1
30 °C	SK	0.85	0.76	1
T0	NP	0.94 ± 0.01	0.70 ± 0.02	3
24 °C	NP	0.98	0.67	1
27 °C	NP	1.00	0.64	1
30 °C	NP	0.88	0.74	1
T0	SK + HA	0.83 ± 0.03	0.80 ± 0.02	3
24 °C	SK + HA	0.80	0.77	1
27 °C	SK + HA	0.87	0.82	1
30 °C	SK + HA	0.87	0.83	1
T0	NP + HA	0.92 ± 0	0.72 ± 0.02	3
24 °C	NP + HA	0.86	0.74	1
27 °C	NP + HA	0.94	0.69	1
30 °C	NP + HA	0.93	0.73	1

For samples greater than *n* = 1, the standard error of the mean is expressed.

## 4 Discussion

This study was conducted to assess the impacts of DOM and temperature on rates of productivity and respiration and involved opportunistic sampling following a series of hurricanes that impacted the southwest Florida coastal region in 2023 and 2024. Our initial hypothesis, that increasing temperature–combined with hurricane-driven inputs of humic acids–would reduce primary production while stimulating respiration processes and DOM degradation, was confirmed. We also confirmed that light dependent respiration, exclusively due to algal respiratory processes, and measurable with ^18^O techniques, was significant compared to microbial community respiration. Warming and DOM enrichment both enhance O_2_ consumption in coastal waters, and respiration is known to increase with elevated DOM loading (Pace et al., 2021). Consistent with this evidence, the experiments reported here following the 2024 hurricane events showed that GPP and NPP declined substantially with warming, and it was even more pronounced in the colored, DOM-rich waters after Hurricane Debby. In contrast, GR rates, solely due to algal respiration and not due to the microbial community as a whole, increased sharply with temperature, frequently exceeding both GPP and NPP. These findings highlight the complex interactions among oceanographic and meteorological processes that influence hypoxia formation with potential implications for *K. brevis* bloom dynamics on the WFS.

### 4.1 Primary production and respiration rates

In the oligotrophic subtropical waters of the WFS, it has previously been shown that daily GPP is often closely balanced by CR, resulting in very low or near-zero NPP under non-bloom conditions (Hitchcock et al., 2000). Those experiments, conducted in Florida in 1996, involved depth-integrated daily primary production rates measured using an earlier iteration of the H_2_^18^O method (Bender and Grande, 1987), while dark CR was measured through dark bottle-light bottle comparative incubations. In that study, dark CR consumed most of the GPP. Contrasting results were later observed by Hitchcock et al. (2010), who investigated community metabolism during *K. brevis* blooms on the WFS. Using diel light and dark O_2_ bottle incubations to estimate NPP and CR, they found that during periods of elevated biomass, NPP became strongly positive, with daytime O_2_ production substantially exceeding respiratory demand. However, that study also did not quantify light-dependent respiration rates. In the study herein, only one sample was collected during a *K. brevis* bloom (~4.0 × 10^5^400 cells L^−1^at NP), and at this station, in contrast to Hitchcock’s findings, as well as across non-bloom stations, GR exceeded both GPP and NPP, particularly at 30 °C.

Consistent patterns in NPP and a tendency toward net heterotrophy have also been observed in other estuaries of Florida even without considering light dependent respiration. Arriola et al. (2025) quantified ecosystem metabolism using dissolved O_2_ measurements from *in situ* probes in Tampa Bay and Biscayne Bay. They estimated vertically integrated GPP, NPP and ecosystem respiration (ER) using the O_2_ mass-balance approach originally described by Odum (1956), where ER is derived from nighttime O_2_ budgets assuming zero GPP, and GPP is calculated from daytime O_2_ budgets assuming constant ER. Their results showed that daily GPP and ER were of similar magnitude, with NPP typically much smaller, indicating that these estuaries operate near metabolic balance with only minor shifts toward autotrophy or heterotrophy. In Tampa Bay, positive NPP was most often associated with periods of higher light availability, increased nutrient supply, and areas benefiting from seagrass recovery and water-quality improvements, while negative NPP occurred more frequently under conditions of elevated terrestrial organic-matter inputs or reduced light. Together, these findings demonstrate that subtropical coastal systems are highly sensitive to relatively small changes in temperature, salinity, light, and organic-matter loading, which can shift ecosystem metabolism toward autotrophy or heterotrophy.

The results of the several different kinds of experiments performed herein following the hurricane events of 2024 presented a consistent pattern: GPP declined substantially with warming, with even stronger reductions observed with temperature increases in the colored, DOM-rich waters after Hurricane Debby. Decreases in GPP with warming may be expected if temperatures exceed the temperature optimum for the phytoplankton community. The light and dark reactions of photosynthesis have different temperature sensitivities and declines at high temperature can be due to denaturing of proteins (Geider, 1987; Glibert, 2024). The NPP rates also decreased with temperature, becoming predominantly negative above 30 °C. In the WFS, temperatures above 30 °C are commonly observed during summer in surface waters (Liu et al., 2006; Li et al., 2022; Nickerson et al., 2023; Sorinas et al., 2023). Rates of GR increased sharply with warming, often exceeding both GPP and NPP. It is well known that respiration is more temperature-dependent than photosynthesis, so that warming disproportionately accelerates respiratory losses relative to carbon fixation (Barton et al., 2020, and references therein). In this study, this was evident not only in the strong temperature dependence of dark respiration, CR, experiments (Figures 7D,E), but also in the light-dependent respiration (i.e., GR) rates that were measured (Figures 5, 6). Previous studies have shown that O_2_ consumption in the light can exceed that in measured in darkness (Bender and Grande, 1987; Kana, 1990). Similar to some of the patterns observed in results reported herein, GR was comparable to, or even exceeded, dark respiration under ambient conditions in several cases, most notably at the TB site after Hurricane Debby (Figure 7A).

Rates of GR also increased with DOM loading when post-Helene rates are compared to those measured pre-Idalia. Such a difference may have been compounded by the reduction in light availability due to high DOM, as the light-dependent experiments pre-Hurricane Debby showed that GR was highest at 60 μmole photons m^−2^ s^−2^, a value about half that used in the subsequent experiments. However, light effects would also be likely to vary with the phytoplankton community (Kana, 1990). Responses also depend on the nature and concentration of the humic material. Depending on the specific nature of the humic substances, these acids can diffuse through algal membranes, where at high concentrations can alter the proton gradient across the inner chloroplast membrane (Allen, 2002). At low concentration there may be stimulation of productivity due to the release of nutrients while at high concentration, photosynthesis may decline, and respiration may increase (Golubkov, 2026). Toropkina et al. (2017) observed, in a study of *Chlorella*, that additions of humic acids led to reduction in photosynthesis and increases in respiration due to the cell’s defense against the production of oxidative stress. Soulié et al. (2022) showed, in a mesocosm experiment, that brownification caused reduction in GPP and CR by almost one third but did not specifically measure GR. These findings highlight that light-driven respiratory processes (e.g., Mehler reaction, cyclic electron flow, photorespiration) represent a substantial portion of O_2_ consumption. Consequently, relying solely on traditional light–dark bottle methods may possibly underestimate respiration rates, particularly under conditions of warming and elevated DOM.

The trends observed in these experiments collectively demonstrate that an increase of only a few degrees in temperature can shift the system toward a more heterotrophic state, especially when combined with storm-driven inputs of organic matter. In this study, temperature was the only variable directly manipulated. However, the comparison among stations after Hurricane Debby, which differed in water color and turbidity, provided insight into how light availability may influence community metabolism. Our results indicate that reduced light availability could affect not only primary production but also light-dependent respiration. Considering that phytoplankton respiration (GR) frequently exceeded production in our experiments, these results indicate that warming and hurricane-enhanced DOM inputs have the potential to increase the development of hypoxic conditions on the WFS. Hurricanes can further influence *K. brevis* bloom dynamics on the WFS via impacts on nutrient delivery: for example, runoff and submarine groundwater discharge were enhanced during the unusually active 2004 hurricane season along the WFS and were hypothesized to support the extensive *K. brevis* bloom during 2005 (Hu et al., 2006). Similarly, the 2018 bloom was associated with substantial flooding and runoff following Hurricane Irma in 2017 (Fritz, 2018). Modeling work by Chen and Li (2025) showed that the strong winds and heavy rainfall associated with Hurricane Ian intensified runoff and stratification on the WFS, first triggering a brief diatom bloom that subsequently transitioned into a prolonged *K. brevis* bloom. Such storms can profoundly reshape nutrient delivery and hydrological conditions, often creating environments favorable for *K. brevis* blooms. Furthermore, studies have associated hypoxic conditions with *K. brevis* blooms in the WFS (Smith, 1975; Heyl et al., 1978; Milbrandt et al., 2021; Turley et al., 2022). Typically, *K. brevis* blooms initiate in early fall (August – September) and terminate in early spring (February – April) (Heil and Muni-Morgan, 2021). However, these blooms can occasionally overwinter and persist into the summer months, even when temperatures exceed their optimal growth range of 22–28 °C (e.g., Vargo, 2009; Ahn et al., 2023; Glibert et al., 2025), indicating that temperatures above 30 °C do not necessarily suppress their blooms. *Karenia brevis* can thermally acclimate, and its photosynthetic and nutritional responses depend not only on prior temperature exposure but also on the N form available (Ahn and Glibert, 2021; Ahn et al., 2023). Only after Hurricane Milton, which occurred during the season when *K. brevis* blooms more typically develop, were substantial abundances of *K. brevis* noted in this study. Together, these examples highlight how hurricane-driven pulses of freshwater, nutrients, and organic matter can set the stage for large and persistent algal blooms in this region. These intense events are expected to become more common, as extreme weather is increasing in intensity and frequency under climate change (Glibert and Li, 2024), and Florida experiences major events roughly every 2 years (Malmstadt et al., 2010).

### 4.2 FDOM

Both Hurricanes Helene and Milton delivered terrestrial humic FDOM that was degraded during the relatively short incubation experiments. The increases in temperature were associated with enhanced biological production of labile, protein-like FDOM and decreases in HIX. Together these results suggest that increasing ocean water temperatures, particularly when coupled with storm-driven terrestrial inputs, can lead to a proportional shift from humified, or terrestrially dominated FDOM to an increasingly labile FDOM pool which can stimulate microbial respiration, thus increasing O_2_ demand and potentially contributing to the development of hypoxia.

Work by Mendoza and Zika (2014) found spatiotemporal variability of humic- and protein-like FDOM along transects beginning at the mouths of the Shark, Caloosahatchee, and Peace Rivers offshore onto the southwest Florida continental shelf. Results showed that increases in humic-like fluorophores delivered via riverine flow, anthropogenic sources such as runoff, and wind driven sediment resuspension correspond with increases in protein-like DOM, indicating increased biological production. In highly urbanized, HAB-prone systems such as Tampa Bay, the timing of these inputs is critical. For example, monthly and seasonal (wet vs. dry) monitoring of urban residential runoff and rainfall entering Tampa Bay revealed shifts in FDOM quality and reactivity. During the wet season, increases in labile FDOM (proteinaceous and microbially derived) were observed concomitant with increased fluxes of DOC and DON which can correspond with the timing of HABs in Tampa Bay waters (Muni-Morgan et al., 2024). The hurricane-driven humic pulses and subsequent production of protein-like FDOM observed in this study fit within a broader picture in which extreme rainfall and storm events define “windows” of enhanced DOM delivery that stimulates respiration and autochthonous production and amplify the effects of warming on O_2_ demand.

As microbial and phytoplankton communities become more metabolically active under warming, greater releases of proteinaceous DOM likely reflect increased exudation, cell turnover, and cellular lysis, which can further reinforce bloom dynamics by fueling heterotrophic activity and nutrient recycling. At site NP, values of BIX were consistently higher than at site SK post Hurricanes Helene and Milton. This indicates greater microbial activity at NP which could be a result of contributions of labile DOM from the surrounding watershed. For example, NP sits at the mouth of Sarasota Bay, which received over 64 million liters of sewage post Hurricane Debby (Farrow, 2024) which preceded Hurricanes Helene and Milton. Conversely, FDOM was more complex and humified at site SK and dominated by terrestrially derived material at T0 which could be due to rainfall generated runoff or riverine inputs. Such a role of precipitation and river discharge in structuring humic-like and protein-like FDOM on the southwest Florida shelf is consistent with the patterns described by Mendoza and Zika (2014). However, increases in relative proportion of protein-like C3 for SK samples more than doubled those in NP. These results suggest that humic compounds at SK stimulated microbial growth by providing nutrients and/or carbon leading to the production of protein-like DOM as a byproduct of metabolism and growth.

After Hurricane Milton, substantial increases in C3 with increasing temperature were observed in the unamended treatments at NP and SK. For example, a percent change exceeding 40% at both NP and SK at 27 °C was observed, highlighting a potential “sweet spot” for DOM processing. This response was markedly lower in the humic acid amended treatments, suggesting that increased humic complexity may constrain autochthonous production of labile DOM, regardless of temperature. However, BIX values decreased at 30 °C in the unamended NP and SK treatments, suggesting a reduction in autochthonous DOM production potentially due to thermal stress or complete exhaustion of labile DOM compounds.

Under stress conditions, an increase in protein-like FDOM is expected, which was evident in our results, especially at higher temperatures. Post-Hurricane Helene, dark CR and light GR rates were higher at SK compared to NP, consistent with the observed increase in the protein-like component (C3) at SK, particularly under elevated temperature conditions. Similarly, post-Hurricane Milton, respiration rates were also higher at SK than NP and increased with temperature. However, in contrast to Helene, the C3 component peaked at 27 °C at both stations (SK and NP), suggesting that the production of protein-like DOM exhibited distinct temperature optima depending on the post-hurricane context or its rapid decomposition at 30 °C.

## 5 Conclusion

Overall, all stations exhibited temperature-driven declines in GPP, a shift toward negative NPP, and increased GR at higher temperatures, demonstrating that warming consistently suppresses photosynthesis while enhancing algal respiratory metabolism. Compared to rates of microbial CR, rates of GR in some cases exceeded, and other cases, nearly matched those of CR. In subtropical coastal ecosystems, where elevated seawater temperatures often coincide with increased terrestrial organic matter inputs (e.g., humic substances), promoting brownification, these metabolic shifts may be further amplified, as shown in the comparison of GR rates post-Helene relative to pre-Idalia rates measured at the same station. These results suggest that warming and hurricane-driven brownification are associated with enhanced algal respiratory metabolism in addition to enhanced CR, highlighting a potential pathway through which climate change could intensify hypoxia in subtropical coastal systems. Since the WFS, like other subtropical coastal ecosystems, integrates both terrestrial and marine influence, it is uniquely vulnerable to the interaction of these multiple stressors. Therefore, studies quantifying how these rates change under different temperatures, and how the addition of humic acids may alter these rates, are essential for understanding the complexity of these processes and how climate change is and will continue to influence them. Furthermore, measuring and incorporating these rates can help improve mechanistic models to better understand how the ocean will change in the future.

## Data Availability

The raw data supporting the conclusions of this article will be made available by the authors, without undue reservation.
