# Supplementary material for: Temperature effects on productivity, respiration, and dissolved organic matter degradation in subtropical Florida waters following hurricane-driven riverine runoff and brownification
Source: Front Microbiol. 2026 Sep 15;17:1884823. doi: 10.3389/fmicb.2026.1884823 (PMC13621497; doi:10.3389/fmicb.2026.1884823)
Supplement: Supplementary file 1 [file Supplementary_file_1.DOCX]

Temperature Effects on Productivity, Respiration, and Dissolved Organic Matter Degradation in Subtropical Florida Waters Following Hurricane-driven Riverine Runoff and Brownification

**Bruna F. Sobrinho^1*^, Amanda Muni-Morgan^2,3^, Cynthia Heil^4^ and Patricia M. Glibert^1^**

^1^ University of Maryland Center for Environmental Science, Cambridge, Maryland, United States

^2^ University of Florida, Wimauma, FL, United States

^3^ Ecosystem Insights LLC, St. Petersburg, Florida, United States

^4^ Mote Marine Laboratory, Sarasota, Florida, United States

Supplementary Material

**Supplementary Table 1** Ambient environmental conditions measured during sampling periods before and after Hurricane Idalia, after Hurricane Helene, and after Hurricane Milton. Environmental parameters include water temperature (˚C), salinity, ammonium (NH_4_^+^, µM), nitrate (NO_3_^-^, µM), dissolved organic carbon (DOC, µM), chromophoric dissolved organic matter (CDOM, absorption at 280 nm), and chlorophyll-*a* (Chl-*a,* µg L^-1^).

| Sample time/condition | Station | Temperature  (˚C) | Salinity | NH_4_^+^  μM | NO_3_^-^  μM | DOC  μM | CDOM  @280 nm | Chl-*a*  µg L^-1^ |
| --- | --- | --- | --- | --- | --- | --- | --- | --- |
| Aug 23, 2023  Pre Idalia | Charlotte Harbor | 29.6 | 23.7 | 1.63 | <0.07 | 686 | 39.5 | 10.08 |
| Sept 9, 2023  Post Idalia | Charlotte Harbor | 28.8 | 16.0 | 3.41 | 2.56 | 1080 | 88.5 | 17.85 |
| Aug 8, 2024  Post Debbie | Tampa Bay  Siesta Key  Caloosahatchee River  Charlotte Harbor | 28  28  28  28 |  |  |  |  |  | 1.84  3.59  2.32  4.31 |
| Oct. 2, 2024  Post Helene | New Pass  Siesta Key | 28  28 |  |  |  |  |  | 10.09  8.55 |
| Nov 20, 2024  Post Milton | New Pass  Siesta Key | 22.7  22.9 | 33.0  31.0 | 3.75  3.86 | 0.19  0.20 |  |  | 4.75  2.86 |

**Supplementary Table 2** Phytoplankton community composition and abundance (cells L^-1^) for samples collected post Hurricanes Debbie, Helene and Milton. “Total other” includes cryptomonads, raphidophytes, and other flagellates.

| **Sampling time** | **Phytoplankton Groups** | **Tampa Bay (TB)** | **New Pass (NP)** | **Siesta Key (SK)** | **Caloosa-hatchee River (CR)** | **Charlotte Harbor (CH)** |
| --- | --- | --- | --- | --- | --- | --- |
| Post Hurricane Debbie | *Karenia brevis* | 0 |  | 0 | 0 | 0 |
|  | Dinoflagellates | 8 × 10^4^ |  | 4 × 10^4^ | 3 × 10^4^ | 0 |
|  | Diatoms | 1.3 × 10^7^ |  | 6.6 × 10^5^ | 6 × 10^4^ | 2.2 × 10^5^ |
|  | Cyanobacteria | 0 |  | 9.6 × 10^5^ | 0 | 6.6 × 10^5^ |
|  | Total other | 2.4 × 10^5^ |  | 6.6 × 10^5^ | 1.8 × 10^5^ | 2.4 × 10^5^ |
| Post Hurricane Helene | *Karenia brevis* |  | 0 | 0 |  |  |
|  | Dinoflagellates |  | 4 × 10^4^ | 8 × 10^4^ |  |  |
|  | Diatoms |  | 4.6 × 10^5^ | 5.4 × 10^5^ |  |  |
|  | Cyanobacteria |  | 0 | 1.1 × 10^7^ |  |  |
|  | Total other |  | 3 × 10^4^ | 1.1 × 10^5^ |  |  |
| Post Hurricane Milton (day 1) | *Karenia brevis* |  | 4 × 10^5^ | 6 × 10^4^ |  |  |
|  | Dinoflagellates |  | 2 × 10^4^ | 0 |  |  |
|  | Diatoms |  | 8 × 10^5^ | 4 × 10^6^ |  |  |
|  | Cyanobacteria |  | 0 | 7 × 10^5^ |  |  |
|  | Total other |  | 4 × 10^4^ | 4 × 10^4^ |  |  |

**Supplementary Figure 1.** Gross respiration (µmol O_2_ (µg Chl-*a*)^-1^ h^-1^) as a function of light (PAR, PAR, μmol photons m*^-2^* s*^-^*^1^) measured in surface waters following Hurricane Idalia.

**References**

Dickson, A. G., Sabine, C. L., Christian, J. R. (2007). *Guide to best practices for ocean CO2 measurements*. PICES Special Publication, 3, 191 pp.
